# Ceramides in Alzheimer's Disease: Key Mediators of Neuronal Apoptosis Induced by Oxidative Stress and A***β*** Accumulation

**DOI:** 10.1155/2015/346783

**Published:** 2015-05-24

**Authors:** Maja Jazvinšćak Jembrek, Patrick R. Hof, Goran Šimić

**Affiliations:** ^1^Laboratory for Molecular Neuropharmacology, Division of Molecular Medicine, Rudjer Bošković Institute, 10000 Zagreb, Croatia; ^2^Department of Psychology, Croatian Catholic University, 10000 Zagreb, Croatia; ^3^Fishberg Department of Neuroscience and Friedman Brain Institute, Icahn School of Medicine at Mount Sinai, New York, NY 10029, USA; ^4^Department for Neuroscience, Croatian Institute for Brain Research, University of Zagreb Medical School, 10000 Zagreb, Croatia

## Abstract

Alzheimer's disease (AD), the most common chronic and progressive neurodegenerative disorder, is characterized by extracellular deposits of amyloid *β*-peptides (A*β*) and intracellular deposits of hyperphosphorylated tau (phospho-tau) protein. Ceramides, the major molecules of sphingolipid metabolism and lipid second messengers, have been associated with AD progression and pathology via A*β* generation. Enhanced levels of ceramides directly increase A*β* through stabilization of *β*-secretase, the key enzyme in the amyloidogenic processing of A*β* precursor protein (APP). As a positive feedback loop, the generated oligomeric and fibrillar A*β* induces a further increase in ceramide levels by activating sphingomyelinases that catalyze the catabolic breakdown of sphingomyelin to ceramide. Evidence also supports important role of ceramides in neuronal apoptosis. Ceramides may initiate a cascade of biochemical alterations, which ultimately leads to neuronal death by diverse mechanisms, including depolarization and permeabilization of mitochondria, increased production of reactive oxygen species (ROS), cytochrome c release, Bcl-2 depletion, and caspase-3 activation, mainly by modulating intracellular signalling, particularly along the pathways related to Akt/PKB kinase and mitogen-activated protein kinases (MAPKs). This review summarizes recent findings related to the role of ceramides in oxidative stress-driven neuronal apoptosis and interplay with A*β* in the cascade of events ending in neuronal degeneration.

## 1. Introduction to A*β*, Lipids, and Oxidative Stress in Alzheimer's Disease

Alzheimer's disease (AD) is the most common neurodegenerative disease and the leading cause of dementia in elderly people. Slow and progressive degeneration of neurons in brain regions involved in learning and memory results in gradual cognitive decline, loss of memory, personality changes, and impairment of normal social and emotional behaviours [1–3]. In spite of major research efforts, current therapeutic approaches do not modify the progression of the disease. Without novel therapeutic improvements, it is estimated that the number of affected people worldwide will rise to 100 million by 2050 [4].

Neuropathologically, AD is characterized by the aberrant accumulation and deposition of misfolded proteins. In particular, extracellular neuritic plaques made of aggregated forms of neurotoxic amyloid *β*-peptide (A*β*) and intracellular deposits of abnormally phosphorylated tau protein are considered as key hallmarks of AD brain [5–7]. Within cell membranes, in unique microdomains called lipid rafts, A*β* is derived from the amyloid precursor protein (APP) through two major steps of enzymatic cleavage [6, 8, 9]. Namely, of particular importance for the pathogenesis of AD is a shift towards the *β*-secretase (BACE1) pathway in which sequential cleavage of APP first by BACE1 and then by *γ*-secretase generates A*β* peptide, a 39- to 43-amino acid fragment. The A*β* peptide, especially A*β*_1–42_, is highly prone to aggregation and self-assembles to form a heterogeneous mixture of oligomers and protofibrils, ultimately deposited as fibrils in senile plaques. Small soluble A*β* oligomers, which correlate strongly with disease onset and severity, are considered as the major neurotoxic species in AD [10–12].

As APP and both *β*- and *γ*-secretase are integral membrane proteins, it comes to no surprise that membrane lipids may regulate subcellular trafficking, activity, and metabolism of these AD related proteins and affect proteolytic processing of APP and generation of A*β* [13]. A deregulation of lipid metabolism has been recognized as an increasingly important factor in the pathogenesis of AD. Numerous studies have shown that alterations in the composition of the brain's lipid profile and cerebral lipid homeostasis are associated with the disease onset and progression [14–17]. In addition, the single most important genetic risk factor for late-onset sporadic AD is the presence of the *ε*4 allele of the apolipoprotein E (apoE), the major brain lipoprotein, which mediates transport of cholesterol and other lipids [18, 19]. Epidemiological and animal studies suggested that a high-fat diet is a serious risk factor for the development of the disease, as it may elevate peripheral cholesterol, perturbate central lipid metabolism, and increase oxidative stress [20–24]. Among different lipid classes, ceramides have attracted much attention in recent years as key contributors in the pathology of AD as they are able to affect both A*β* generation and tau phosphorylation [25, 26]. Alterations in sphingolipid metabolism that result in accumulation of long-chain ceramides have been observed in normal aging and in the brains from AD patients [27]. Furthermore, ceramide- as well as other lipid-lowering drugs may reduce accumulation of A*β in vitro* and* in vivo*, providing novel promising avenues for improving therapeutic effectiveness in AD [28–32].

In addition to increased ceramides, the pathogenesis of AD is also tightly linked to increased oxidative stress. Oxidative stress is accompanied with mitochondrial dysfunction, pronounced inflammation, gliosis, axonal degeneration, and impairment of synaptic transmission that ultimately end in progressive neuronal loss, predominantly by apoptosis. Neuronal loss is particularly evident in vulnerable brain areas such as entorhinal cortex, hippocampal formation, and association regions of neocortex [33–37]. Evidence suggests that a long quiescent period of progressive oxidative injury precedes and actually leads to the seemingly sudden appearance of clinical and pathological symptoms of the disease [38]. A high-fat diet may play an important role in accelerating an age-related increase in oxidative stress and induce changes in energy metabolism and brain function that promote the development of cognitive alterations including dementia [23, 39]. Moreover, decreased antioxidant response may additionally contribute to the high-fat diet-induced exacerbation of cognitive dysfunction, tightly implicating oxidative stress as an important mediator in the impairment of cognitive functions [23].

## 2. Ceramides in the Pathogenesis of AD

Ceramides are central molecules of sphingolipid biosynthesis and catabolism. Sphingolipids represent a heterogeneous class of lipids that contains backbone of sphingoid bases, a set of aliphatic amino alcohols of which sphingosine is the most common in mammals [40]. Sphingolipids are particularly abundant in aforementioned lipid rafts, highly dynamic membrane microdomains that regulate spatial and temporal assembly of signalling and trafficking molecules, acting as platforms for signal transduction [10, 41]. Hence, beside important role as structural components of eukaryotic membranes, particularly in the outer leaflet of the plasma membrane, sphingolipids and their metabolites act as second messengers in diverse signalling pathways. These transduction pathways determine differentiation, cell growth, growth arrest, and apoptosis, depending on the levels and species of metabolites formed, cell and receptor types, and downstream targets [17, 30, 42, 43]. Although sphingolipids represent only a small fraction of the total cellular lipid pool, even subtle disturbances in their balance may contribute to the development of neurodegenerative diseases [44–46]. For example, levels of sphingomyelin, the most abundant sphingolipid in mammalian cells, are increased exclusively in the cerebrospinal fluid of patients in prodromal AD [47], while an increased ratio of sphingomyelin to ceramides is considered as a potential blood-based marker of the disease progression [48].

Ceramides, as primary metabolic products of sphingomyelin catabolism and the major bioactive molecules of sphingolipid pathways, also comprise a heterogeneous class of lipids. They are composed of sphingosine attached to different fatty acids varying in length from 14 to 26 carbon atoms. Most commonly, sphingosine is attached to palmitic (C16) and stearic (C18) non-hydroxy fatty acids [43, 49, 50]. Beside their essential role in signal transduction, they also act as regulators of synaptic function, contributing to the maintenance of synapses, dendritic spines, and neuronal transmission in association with other sphingolipids and cholesterol in lipid rafts [43, 44, 51].

**Figure 1:**
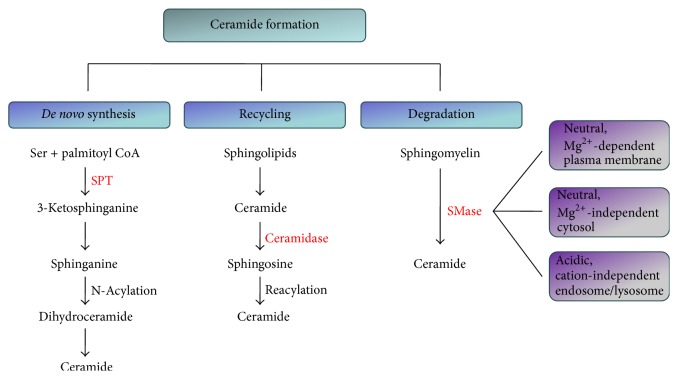
Ceramide formation via* de novo* synthesis, recycling, and degradation. In the catabolic pathway, ceramides are generated through sphingomyelin hydrolysis by sphingomyelinases (SMases). In* de novo* synthesis, serine palmitoyltransferase (SPT) is the rate-limiting enzyme. In the recycling pathway, sphingosine, the product of sphingolipid catabolism, is salvaged through reacylation, resulting in ceramide production.

Ceramide formation occurs via three different pathways:* de novo* synthesis, recycling, and degradation (Figure 1). In the catabolic pathway, ceramides are generated through the hydrolysis of phosphodiester bonds in sphingomyelin by sphingomyelinases (SMases). SMases are sphingomyelin-specific forms of phospholipase C enzymes, located either in lysosomes or at the plasma membrane. Several isoforms of SMases have been identified that differ in optimal pH range, subcellular localization, and cation dependence. In particular, a Mg^2+^-dependent neutral SMase, the most prominent form, is found at the plasma membrane and a Mg^2+^-independent neutral SMase is localized in the cytosol, while a ubiquitous cation-independent acidic SMase is active in the endosomal-lysosomal compartments [43, 52]. Unusually, the gene for acidic SMase generates two unique products through the differential trafficking of a single-protein precursor. One of these products is commonly studied and already mentioned lysosomal form, while the other, an alternative acidic SMase, is secreted extracellularly where it potentially may participate in the hydrolysis of sphingomyelin in the outer leaflet of the plasma membrane and bloodstream lipoproteins [42, 53]. Because neutral and acidic SMases can be activated rapidly and transiently in response to diverse exogenous and endogenous stimuli, they are considered major pathways for ceramide generation in early transduction [54]. Both neutral and acidic SMases are active in neurons, but neutral SMases are localized in axons, while acidic SMases are found predominantly in the neuronal perikarya [30].

Ceramides can also be synthesized by a* de novo* endogenous biosynthetic pathway that begins with the condensation of serine and palmitoyl CoA, a reaction catalyzed by the rate-limiting enzyme serine palmitoyltransferase (SPT), to form 3-ketosphinganine. This moiety is further reduced to the sphingoid base sphinganine, which is subsequently N-acylated to form dihydroceramide, and finally after introduction of a double bond to the position C4 of the dihydroceramide, the mammalian type ceramides are formed [42, 43]. At last, ceramides are generated in an alternative pathway that relies on the reverse activity of the enzyme ceramidase and is known as the salvage pathway as catabolic fragments are reused for biosynthesis. In this recycling pathway, complex sphingolipids are broken down to ceramide and then by ceramidase to sphingosine, which is then reconverted by reacylation to yield ceramide [49, 55].

As previously mentioned, ceramides have been suggested to be key players in the pathogenesis of AD. Elevated basal serum levels of particular ceramide species are associated with a higher risk for developing AD [56]. Increased ceramide levels are also present in brains of AD patients in comparison to age-matched neurologically normal control subjects [25, 57, 58]. Importantly, levels of ceramides are found increased in patients with mild to moderate symptoms, indicating that alterations in lipid metabolism occur in the early stages of AD progression [27]. In addition, it appears that serum ceramides vary according to the time of the onset of memory impairment [59]. Furthermore, aging itself is accompanied by a gradual increase in brain A*β* content that can be reduced by inhibitors of neutral SMase [30, 60]. Gene expression abnormalities of enzymes involved in sphingolipid metabolism are observed at different stages of AD progression. In particular, expression of genes related to ceramide homeostasis is altered in a way that enzymes participating in the* de novo* synthesis of ceramide and in sphingomyelin hydrolysis are increased as a function of the disease progression [58, 61]. Alterations in sphingolipid metabolism during normal brain aging and in the brain of AD patients that result in accumulation of long-chain ceramides may contribute to neurotoxic action of A*β* and exacerbate progression of the disease (see below).

At higher concentrations ceramides promote cellular dysfunction and act as regulatory mediators of programmed cell death [62]. Short-chain cell-permeable ceramide analogues (C2-ceramide and C6-ceramide) have been extensively used in studies aimed at elucidating ceramide-mediated signalling death pathways and their role in neurodegeneration [63–66]. A specific sphingomyelin synthase SMS1-related enzyme has been discovered that functions as a regulator of ceramide homeostasis, probably operating as a ceramide sensor aimed at protecting cells against ceramide-induced cell death [42, 67]. As phosphorylated derivatives of ceramides, such as sphingosine-1-phosphate, generally stimulate proliferation and exhibit antiapoptotic properties, at least in part by regulating different family members of mitogen-activated protein kinases (MAPKs), very small shifts in ceramide metabolism may determine neuronal fate in response to a variety of stimuli [66, 68, 69].

There are numerous downstream targets of ceramides. Evidence suggests that ceramides act either by changing the organization of cellular membranes or by direct binding and activation of target proteins. Thus, directly or indirectly, ceramides regulate MAPKs, protein kinase C*ζ* (PKC*ζ*), ceramide-activated protein kinases (CAPK), ceramide-activated protein phosphatases (CAPP), cathepsin D, and diverse phospholipases [43, 70].

## 3. Ceramides and Oxidative Damage in AD

Several lines of evidence pointed out that generation of free radicals is one of the major contributors to the neurotoxic effects of ceramides and A*β* in AD [27, 71, 72]. Increase in ceramide levels stimulates generation of ROS in a concentration-dependent manner, establishing a link between oxidative stress and sphingolipid metabolism with detrimental consequences for neuronal survival [70]. In neuronally differentiated PC12 cells, it is shown that mitochondrial ROS production is an early step in the activation of the apoptotic sphingomyelin-dependent transduction pathway, followed by translocation of the transcription factor NF-*κ*B. Complex 1 of the mitochondrial electron transport chain has been identified as the origin of free radicals. Antioxidant treatment abolished ROS production almost completely and prevented both the NF-*κ*B translocation and neuronal death [73, 74]. Another study on PC12 cells demonstrated almost simultaneous increases in ROS, [Ca^2+^]_cyt_ and [Ca^2+^]_mit_ in ceramide-mediated cell death but found that only the latter was directly implicated in reduced survival [75]. In hippocampal primary cultures containing predominantly astrocytes, ROS levels were also increased in ceramide-treated cells in a dose-dependent manner, as indicated by the amount of measured hydrogen peroxide and upregulated Ddit3 expression, an indicator of cellular oxidative stress [76].

As mentioned before, oxidative stress-induced neuronal damage is one of the most prominent characteristics of AD [36, 77–79]. Oxidative stress occurs when cellular antioxidant defences are insufficient to keep the levels of ROS below a toxic threshold. It has deleterious effects on cellular macromolecules, including proteins, lipids, carbohydrates, and nucleic acids, causing their structural and functional alterations, and frequently leading to cell death [78–82]. The brain is particularly vulnerable to oxidative damage due to its high rate of oxygen consumption, high production of ROS, limited oxidative defences, low repair mechanisms activity, and large amount of polyunsaturated fatty acids (PUFAs) [81, 83]. PUFAs are highly prone to ROS-induced lipid peroxidation, a chain reaction of free radical formation in the lipid parts of cellular membranes. Lipid peroxy radicals may change fluidity and permeability of cellular membranes or may directly attack and damage intracellular biomacromolecules. 4-Hydroxynonenal (HNE) and malondialdehyde and acrolein are highly toxic typical end-products of lipid peroxidation. HNE is considered to be the most toxic aldehyde. As a strong electrophile, it readily reacts with thiol and amino groups of cysteine, histidine, and lysine, leading to protein damage [84–86].

The role of lipid peroxidation has been repeatedly emphasized as one of the major causes of oxidative damage in AD [35, 87]. When cultured neurons were pretreated with *α*-tocopherol (a strong antioxidant) before exposure to A*β*, amounts of HNE adducts and long-chain C:18 and C:24 ceramides were significantly lower than in A*β*-treated neurons, while their survival was enhanced [27]. Accumulation of HNE is associated with brain aging, and levels of HNE increase in parallel with AD severity [27, 88, 89]. Moreover, membrane-associated oxidative stress occurs together with alterations in the composition of brain lipids. In the postmortem brain increase in HNE is accompanied with depletion in PUFAs [90]. Epidemiological studies suggest that increased intake of *ω*-3 PUFA, such as docosahexaenoic acid (DHA), reduces the risk of AD and decreases A*β* plasma levels [91, 92]. Animal studies also indicated that dietary DHA could protect against A*β* production, accumulation, and downstream toxicity and improve cognitive performance. Interestingly, the largest reduction in overall plaque burden was detected in the hippocampus and parietal cortex, the most affected regions in AD [93, 94]. In contrast, levels of HNE protein adducts, HNE-lysine and HNE-histidine, were significantly elevated in the hippocampus of rats with diet-induced hyperlipidemia [24]. In addition to adduct formation, toxic effects of HNE are also related to impairment of glucose transport and subsequent ATP depletion [95]. Namely, abnormal metabolic changes, including pronounced decrease in glucose uptake and metabolism, are also characteristic for AD brain (for review see [96–99]). The presence of other markers of oxidative injury, such as protein nitration, reactive carbonyls, and DNA oxidation, together with marked decline in oxidation-sensitive enzymes, was also confirmed in the brain and cerebrospinal fluid of AD patients by immunohistochemical and histopathological studies [38, 77, 90]. Moreover, a general reduction in thioredoxin levels has been observed in AD brains [100], further supporting the role of oxidative mechanism in the pathogenesis of AD.

Related to oxidative stress, it is of particular relevance that elevated levels of copper are present both in brain and plasma of AD patients [101–103]. In the presence of biological reductants, Cu^2+^ can be reduced to Cu^+^, catalyzing the formation of highly toxic and extremely reactive hydroxyl radicals via Fenton chemistry [104, 105]. Besides generating ROS, excess copper may induce alterations in APP processing, increase production of A*β*, and impair learning and memory in animal models [106, 107].

Interestingly, enhanced levels of Cu^2+^ trigger apoptosis via activation of acidic SMase and release of ceramides in hepatocytes [108]. Although it has yet to be confirmed, this finding might indicate similar association between copper-induced oxidative stress, SMase activation, and ceramide increase in neuronal cells. Lipid soluble ceramides generated by excess hepatic production readily cross the blood-brain barrier. Lyn-Cook et al. provided supportive evidence that hepatic ceramide and proinflammatory cytokine production play important roles in the pathogenesis of cognitive impairment and AD-type neurodegeneration by inducing oxidative stress in the brain, as evidenced by increased HNE [109].

Oxidative stress also mediates A*β*-toxicity that might be reversed by preincubation with antioxidant* N*-acetyl-L-cysteine, a glutathione precursor [110]. Besides, A*β* indirectly increases ceramides through oxidative stress-mediated mechanisms [27]. A recent study by Giraldo et al. suggested that, in addition to causing damage because of the action of free radicals, oxidative stress deranges signalling pathways, in particular p38 MAPK pathway, ending in A*β*-induced tau hyperphosphorylation [111].

### 3.1. Role of Ceramides in Neuronal Apoptosis

**Figure 2:**
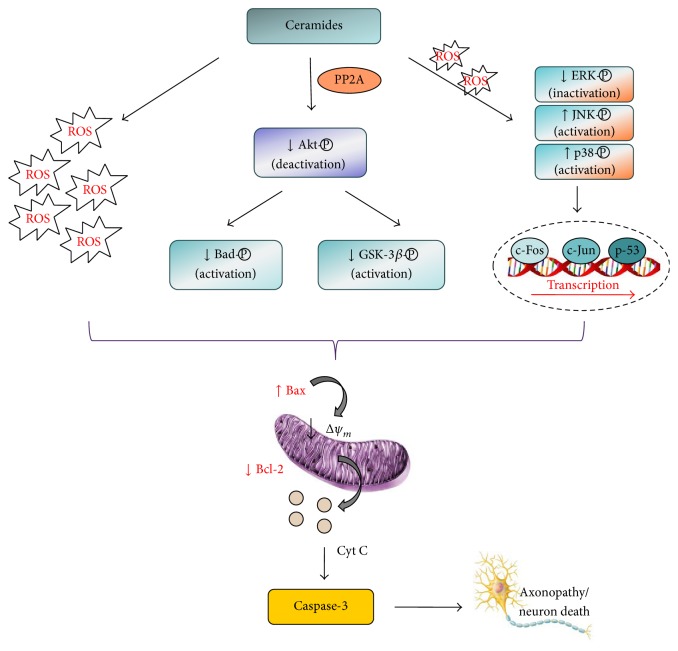
Role of ceramides in neuronal apoptosis. Overexpressed ceramides may provoke apoptosis by directly participating in ROS generation, through modulation of mitogen-activated protein kinases (MAPKs) signalling pathways, and by inhibition of prosurvival phosphatidylinositol 3-kinase (PI3-K)/Akt pathway via protein phosphatase 2A (PP2A) dephosphorylation, leading to activation of Bad and glycogen synthase 3*β* (GSK-3*β*). These cascades of biochemical alterations ultimately lead to neuronal death by diverse mechanisms, including depolarization and permeabilization of mitochondria (Δ*ψ*_*m*_), Bcl-2 depletion and Bax increase, cytochrome c release, and caspase-3 activation.

Incidence of apoptosis is elevated in analysis of postmortem AD brains [112–114]. Overexpressed ceramides may directly participate in the generation of free radicals in mitochondria and provoke mitochondrial dysfunction and apoptosis (Figure 2). A recent study has confirmed that ceramide-induced mitochondrial failure may lead to oxidative stress and poly(ADP-ribose) polymerase-1 (PARP-1) activation, a characteristic biochemical hallmark of apoptosis [115]. Ceramides might initiate a cascade of detrimental alterations that ultimately lead to apoptotic cell death by diverse mechanisms, including depolarization and permeabilization of mitochondria, increased production of ROS, cytochrome c release, Bcl-2 depletion, caspase-3 activation, and inactivation of Akt followed by dephosphorylation of Bad [116–118].

In particular, the cell-permeable C2-ceramide and C6-ceramide induce cytochrome c release in isolated mitochondria [119] and primary cortical neurons [117]. Ceramides do not trigger direct cytochrome c secretion or release but enhance permeability of the mitochondrial outer membrane via ceramide channel formation that allows release of all intermembrane space proteins with a molecular weight up to 60 kDa, including cytochrome c [120, 121]. Beside cytochrome c, ceramides induce translocation of other proapoptotic mitochondrial proteins such as Omi, second mitochondrion-derived activator of caspase (Smac), and apoptosis-inducing factor (AIF) [122]. Formation of these ceramide channels is sensitive to the free ceramide concentration suggesting that alterations in ceramide metabolism induce changes in channels assembling and disassembling, regulating the permeability of the outer mitochondrial membrane. Opening of the permeabilization transition pore complex, that results in mitochondrial swelling, rupture of mitochondrial membranes, and release of cytochrome c, may represent an additional mechanism mediating apoptotic cytochrome c release from the intermembrane space of mitochondria into the cytosol [123, 124]. A decrease in mitochondrial membrane potential is a marker of the opening of the transition pore complex and has been observed in ceramide-induced apoptosis of primary cortical neurons [117, 125]. Similarly, in cerebellar granular cells, C2-ceramide affected mitochondrial integrity and induced a decrease in mitochondrial membrane potential as well as a massive release of cytochrome c [126].

Following release, cytochrome c binds to the apoptotic protease activating factor-1 (APAF-1) and activates the caspase cascade [127]. Thus, neuronal death induced by ceramides is often associated with the mitochondrial pathway regulated by caspase-9/caspase-3, as observed in cortical neuronal cultures and SH-SY5Y human neuroblastoma cells [116, 117, 125, 126]. In neuroblastoma SK-N-MC cells,* N*-acetyl-sphingosine (C2-ceramide) also induced caspase-3-dependent apoptosis as evidenced by DNA fragmentation and chromatin condensation [128]. Furthermore, ceramide-induced increase in mitochondrial calcium was responsible for activation of caspase-8, cytochrome c release, and death of nerve growth factor- (NGF-) differentiated PC12 cells [75]. Experiments on SH-SY5Y cells indicate that inhibition of caspases is not sufficient to block ceramide-induced cell death completely due to the translocation of AIF from mitochondria to the nucleus, activation of PARP-1, and accumulation of poly (ADP-ribose), thus supporting the involvement of caspase-independent programmed cell death in ceramide-induced injury [70, 129].

Ceramides may act through a variety of signalling pathways to modulate cell death. In cortical and HMN1 motor neuron cells, exposure to ceramides induces serine-threonine kinase Akt dephosphorylation and deactivation followed by dephosphorylation and activation of proapoptotic regulators such as Bad. Furthermore, exposure to ceramides induces dephosphorylation and activation of glycogen synthase kinase 3*β* (GSK-3*β*), one of the key enzymes participating in the phosphorylation of tau protein and also involved in control of apoptosis [117, 130–132]. Reduction of intrinsic Akt activity is associated with mitochondrial depolarization and permeabilization, cytochrome c release, and caspase-3 activation [117]. A recent study on SH-SY5Y cells also indicated that ceramide inhibits PI3-K/Akt pathway leading to lower phosphorylation and activation of GSK-3*β* and Bad as downstream targets, decreases antiapoptotic Bcl-2, and increases proapoptotic Bax mRNA/protein levels [70]. C2-ceramide-induced increase in Bax levels leads to formation of Bax homodimers, mitochondrial permeabilization, and finally neuronal death [125, 126]. A potent endogenous antioxidant S-nitrosoglutathione, a NO carrier able to terminate oxidative stress in the brain via a number of different mechanisms, can neutralize A*β*- and ceramide-induced toxicity in primary cortical cultures through multiple prosurvival signalling cascades, including the activation of cGMP/PKG and PI3-K/Akt pathways. Activation of these signalling pathways ends in the induction of antiapoptotic and antioxidative proteins such as Bcl-2 and thioredoxin [118]. Negative regulation of kinase Akt through dephosphorylation and concomitant inhibition of antiapoptotic and prosurvival Akt signalling is also observed in differentiated PC12 cells after treatment with C2-ceramide and determined to be the result of enhanced Akt dephosphorylation by ceramide-activated protein phosphatases (CAPP) [133]. In particular, the serine-threonine protein phosphatases 1 (PP1) and 2A (PP2A) act as CAPP and exert important role in ceramide-mediated neuronal death [134–136]. Additional mechanisms responsible for ceramide-induced neuronal death may involve dephosphorylation and inactivation of Bcl-2 after activation of mitochondrial PP2A [137]. Namely, Bcl-2 as a downstream target in Akt signalling loses its antiapoptotic properties after ceramide-induced dephosphorylation. Moreover, PP2A indirectly provokes activation of GSK-3*β* via the PI3-K/Akt pathway, and GSK-3*β* in turn might regulate caspase-2 and caspase-8 in ceramide-induced apoptosis [131]. Overexpression of active PKB/Akt or Bcl-2 successfully prevented neuronal death in ceramide-induced caspase-independent apoptosis related to AIF translocation and PARP activation [129]. However, it should be mentioned that, despite general beneficial role of Akt in neuronal survival, increased Akt activation and hyperphosphorylation of critical Akt substrates have been observed in AD brain, together with a significant loss and altered distribution of phosphatase and tensin homologue deleted in chromosome 10 (PTEN), the major negative regulator of Akt. Such findings support the involvement of aberrant control of Akt and PTEN signalling in AD and suggest that treatments aimed at activating the particular pathway in AD need to be considered carefully [138, 139].

In addition to Akt kinase, downstream ceramide targets are c-Jun N-terminal kinases (JNK) that mediate cell death in AD [140]. Increased ceramide levels have been associated with JNK activation via phosphorylation in differentiated PC12 cells [141]. The JNK-inhibitor L-JNK1 inhibited the stimulatory effects of C2-ceramide on caspase-9 and caspase-3 and prevented C2-ceramide-evoked cell death in cerebellar granule cells [126]. Transcription factor c-Jun is the probable target of JNK signalling in ceramide-evoked death as increased c-Jun phosphorylation was observed in neuronal nuclei following exposure to ceramide. Moreover, it seems that p38 and JNK/c-Jun pathways cooperate to induce neuronal apoptosis [142]. It is demonstrated that ceramides decrease phosphorylation of extracellular signal-regulated kinases (ERKs) and their upstream activators MAPKs kinases (MEKs) and trigger JNK and p38 MAPK cascades inducing neuronal death through caspase-3 activation and upregulation of c-jun, c-fos, and p53 [122, 143]. In another study on SH-SY5Y cells, inhibitors of ERK1/2 and JNK signalling, UO126 and SP600125, respectively, prevented cell death evoked by C2-ceramide confirming the role of these signalling pathways in neuronal death [70]. Similarly, in primary cortical neurons p38 and ERK1/2 inhibitors blocked ceramide-activated apoptotic signalling upstream of mitochondria, by attenuating release of prodeath effectors and reducing caspase-3 activation [122].

### 3.2. Role of A*β* in Neuronal Apoptosis

Similar to ceramides, A*β* induces neuronal apoptosis in the brain, in transgenic mice, and in neuronal cell cultures [25, 144–149]. In particular, A*β* depletes Ca^2+^ in endoplasmic reticulum that results in cytosolic Ca^2+^ overload, decrease in mitochondrial membrane potential, Bax translocation to mitochondria, oxidative damage of mitochondrial DNA [150], and activation of mitochondrial (intrinsic) apoptotic pathway [151]. Evidence also exists for the activation of extrinsic apoptotic pathway in A*β*-evoked death. In cortical neurons exposed to A*β*, activated JNK is required for the phosphorylation and activation of the transcription factor c-Jun that in turn stimulates transcription of several target genes, including the Fas ligand. Following binding of Fas ligand to its Fas receptor, a cascade of events leads to caspase activation and ultimately apoptotic death [147]. Neuronal death provoked by A*β* is preceded by selective alterations in the expression of the Bcl-2 family of apoptosis-related genes. In one study A*β* significantly reduced expression of antiapoptotic Bcl-w and Bcl-x_*L*_. Accordingly, neuronal death was significantly increased by Bcl-w suppression but significantly reduced by Bcl-w overexpression. Downstream of Bcl-w, A*β*-provoked apoptosis was characterized by mitochondrial release of Smac, while JNK turned out to be the upstream mediator of A*β*-induced Bcl-w downregulation and Smac release [152]. A*β*-induced downregulation of Bcl-2 has also been observed, together with increased Bad phosphorylation [149]. In oligodendrocytes, A*β*-induced apoptosis following cytochrome c release involves activation of neutral SMase and increased ceramide accumulation, upregulation of death protein 5 (DP5/Hrk), a BH3-only proapoptotic protein from Bcl-2 family of apoptotic regulators, and also JNK activation [153]. Upregulation of Hrk gene following ceramide exposure was also confirmed in neuroblastoma SH-SY5Y cells [70]. In addition, it is demonstrated that substances acting along the PI3-K/Akt pathway provide strong neuroprotection against A*β*-induced toxicity [154–157]. Interestingly, analyses in APP transgenic mice revealed a significant decrease in hippocampal Akt phosphorylation at 13 months, at a time that corresponds with plaque appearance and memory impairments in these mice [158].

### 3.3. Interplay between Ceramides, A*β* and Oxidative Stress

As already mentioned, sphingomyelinases have a prominent role in sphingolipid metabolism and ceramide formation [159, 160]. Numerous studies have indicated a link between SMase activation and oxidative stress. It was shown that H_2_O_2_-induced alterations in redox homeostasis and activation of neutral sphingomyelinase (N-SMase) result in accumulation of proapoptotic ceramides in neuronal PC12 cells [161]. In human primary neurons it was also demonstrated that toxic oxygen species such as superoxide and hydrogen peroxide can induce N-SMase activity indicating that ceramide production is redox-sensitive [162]. Recently, study of Dotson II et al. confirmed that neutral SMase-2, the major SMase activated in inflammation and during oxidative stress, is indeed the redox-sensitive enzyme and resolved that its basal activity is influenced by antioxidant enzyme thioredoxin [163]. Furthermore, expression of both acidic and neutral SMase was found upregulated in patients with AD in comparison with age-matched controls [25, 58, 164]. Even in normal aging, a membrane-associated N-SMase activity is upregulated in a brain region-specific pattern, particularly in hippocampus and striatum [165]. Altered sphingomyelin metabolism due to increased activity of acidic and neutral SMases, in combination with induced oxidative stress, ultimately provokes synaptic dysfunction, induces apoptosis, and contributes to disease pathogenesis [25, 63].

**Figure 3:**
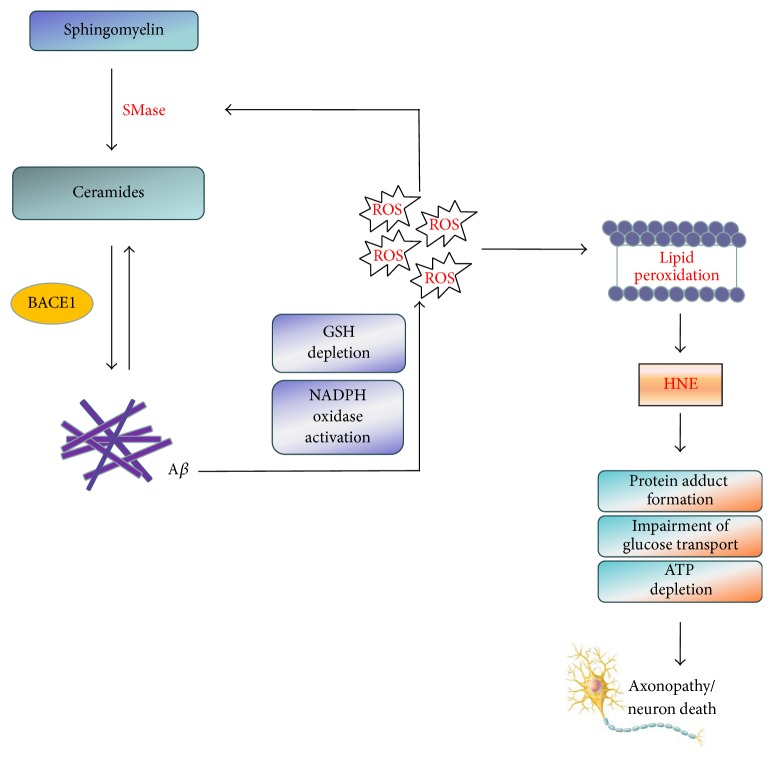
Interplay between ceramides, A*β* and oxidative stress. Increased levels of ceramides directly increase levels of A*β* by increasing the half-life of BACE1 through posttranslational stabilization. As a positive feedback loop, the generated A*β* induces a further increase in ceramide levels by activating SMases activities in a ROS-dependent fashion. A*β* promotes accumulation of free radicals via NADPH oxidase activation and glutathione (GSH) depletion and induces membrane oxidative stress leading to generation of 4-hydroxynonenal (HNE), the neurotoxic product that exacerbates neuronal death through protein adduct formation, impairment of glucose metabolism, and ATP depletion.

*In vitro* and* in vivo* studies have revealed interactive network of processes establishing the link between levels of ceramides and A*β* generation and aggregation (Figure 3). In particular, biogenesis of A*β* is regulated by the endogenous pool of ceramides. In fact, evidence exists that membrane-associated oxidative stress and ceramide production are required for A*β*-induced neuronal death [27]. Namely, pretreatment of neurons with ISP-1 (an inhibitor of serine palmitoyltransferase) results in a significant decrease in the number of neurons killed by A*β*_1–42_, suggesting a pivotal role of ceramide production in A*β*-induced neurotoxicity. Exogenous addition of ceramide mimetics and increased levels of endogenous ceramide induced by N-SMase treatment directly increased level of A*β* by affecting *β*-, but not *γ*-, cleavage of APP [64]. Synthetic ceramide analogues also may affect processing of APP to generate A*β* [166]. Processing of APP to A*β* peptide occurs predominantly in lipid rafts and BACE1 is considered as the rate-limiting enzyme in this process [167]. Membrane ceramides embedded in lipid rafts facilitate production of A*β* by increasing the half-life of *β*-secretase (BACE1) through posttranslational stabilization [64]. Additionally, membrane lipids could directly interact with the components of *γ*-secretase complex or APP via their hydrophobic moieties or polar head groups and underlie the effects on sphingolipids on secretase activity [13]. Recently, it was shown that synthetic ceramide analogues may function as *γ*-secretase modulators that increase A*β*_42_ production, emphasizing important effect of the lipid environment on *γ*-secretase activity [168]. Ceramide also mediates A*β*-induced death in oligodendrocytes. A*β*-provoked activation of N-SMase and generation of ceramide can be inhibited by antioxidant* N*-acetylcysteine, a glutathione (GSH) precursor, indicating that activation of N-SMase-ceramide pathway in glial cells involves oxidative stress after depletion of cellular GSH stores [169].

In addition, ceramides may affect A*β* production by an alternative mechanism. During aging the brain switches from using high-affinity tropomyosin-related kinase A (TrkA) to low-affinity p75 neurotrophin receptor (p75NTR) as the main receptor for nerve growth factor (NGF). Both receptors regulate processing of APP. While TrkA decreases *β*-cleavage of APP, p75NTR enhances APP processing. Ceramide accumulation increases the interaction between p75NTR, NGF, and ceramides and activates *β*-secretase pathway in APP processing and A*β* generation [60].

Soluble and fibrillar forms of A*β* generated by ceramide increase can activate both neutral and acidic SMases pointing out that ceramide production induces a vicious cycle in which initial ceramide formation leads to more ceramide accumulation via A*β* [170, 171]. Experiments performed on human primary neurons and oligodendrocytes support a more prominent role of N-SMase in A*β*-induced toxicity [162, 169]. Similarly, injection of fibrillar A*β* is capable of inducing SMase activity and raising ceramide levels in mouse hippocampus and neocortex 7 days after administration [172]. A*β*-induced production of ceramides is redox-sensitive as ROS are involved in the activation of N-SMase. A*β* induces NADPH oxidase-mediated production of superoxide radicals that are further converted to hydrogen peroxide and involved in the activation of N-SMase [162]. Moreover, A*β*-mediated activation of SMases and accumulation of ceramide can be inhibited by antioxidant molecules such as GSH precursors and *α*-tocopherol and increased by GSH depleters, implying that A*β* activates SMase-ceramide cascade via an oxidative mechanism [27, 162, 169, 171]. Furthermore, A*β* may decrease levels of GSH, the major antioxidant defence system against toxic oxygen species, thus promoting accumulation of ROS [146, 151, 173]. In turn, GSH depletion activates N-Smase activity [159, 174]. Besides, experiments in rat cortical cultures demonstrated that bacterial SMase decreases the expression of thioredoxin-1, another redox protein with potent antioxidative properties, whose decreased expression might have detrimental consequences on neuronal survival [65, 149].

As emphasized before, the ability of A*β* to raise ceramide content is particularly associated with increased levels of membrane oxidative stress. A*β* is inserted as oligomers into the bilayer and serves as a source of ROS, thus inducing oxidative injury primarily as a result of increased lipid peroxidation [36, 77, 175]. The magnitude of brain lipid peroxidation is closely related to the extent of neurodegeneration in AD [27, 88, 89, 176]. Evidence suggests that HNE acts as predominant mediator of toxic effects of A*β* [177]. Remarkable increases in level of lipid peroxide products, together with enhanced SMase activity and ceramide content, have been observed in time- and region-specific manner in rat brain after single injection of A*β* [172]. Cutler et al. suggested that lipid peroxidation is sufficient to induce alterations in ceramide levels in neurons exposed to A*β*, as similar increase in levels of ceramides occurs in neurons exposed to iron that also catalyzes production of hydroxyl radicals and membrane lipid peroxidation [27]. Interestingly, formation of HNE adducts occurs more frequently in the brains of AD patients who carry the apoE *ε*4 allele [178]. Moreover, inhibition of serine palmitoyltransferase, the rate-limiting step in sphingolipid synthesis, prevents HNE increase and attenuates A*β*-induced neuronal death [27].

### 3.4. Interplay between Neurons and Astroglia in Sphingolipid Metabolism

**Figure 4:**
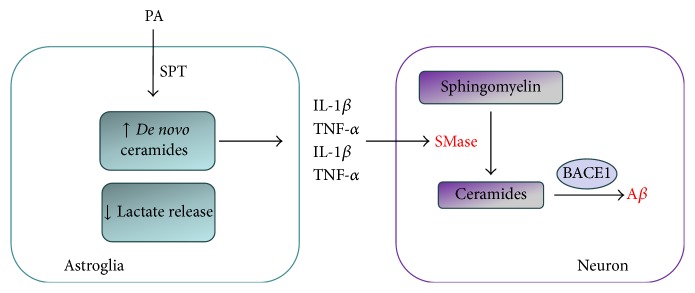
Interplay between neurons and astroglia in sphingolipid metabolism. Conditioned medium from palmitic acid- (PA-) treated astrocytes upregulates neuronal *β*-secretase (BACE1) leading to A*β* accumulation. PA increases* de novo* synthesis of ceramide and downregulates glucose uptake in astroglia. PA-induced activation of serine palmitoyltransferase (SPT) leads to production of proinflammatory cytokines, which after release in surrounding media activate neuronal sphingomyelinases (SMases), increase the ceramide pool, and ultimately lead to increase in A*β* accumulation.

Recent studies have revealed elaborated communication between neurons and glia related to sphingolipid metabolism (Figure 4). Thus, deregulation of ceramide metabolism in astrocytes may induce A*β* secretion, as well as tau phosphorylation, in neurons. Namely, in primary rat cortical neurons treated directly with pathological concentrations of saturated free acid, palmitic acid (PA), there was no change in BACE1 level. On the contrary, conditioned medium from PA-treated astrocytes effectively upregulated neuronal BACE1, with consequent increase in APP processing and accumulation of the C-terminal fragment of APP. As cotreatment with antioxidant 1,3-dimethylurea attenuated PA-induced upregulation of BACE1, it is concluded that PA increases amyloidogenesis through astroglia-mediated oxidative stress [179].

Further studies revealed that PA increases* de novo *synthesis of ceramide in astroglia, which in turn induces production of A*β* in neurons. Inhibition of astroglial-ceramide synthesis by L-cycloserine, an inhibitor of serine palmitoyltransferase (SPT), prevented the PA-astroglia-induced BACE1 upregulation and secretion of A*β* [180]. The same results are obtained in TgCRND8 mice, an early-onset AD model encoding the double mutant form of the APP695. After subcutaneous administration of L-cycloserine, cortical A*β*_42_ and hyperphosphorylated tau levels were downregulated in these mice, in parallel with the inhibition of SPT and ceramide content [181].

In addition, PA may decrease levels of astroglial glucose transporter and downregulate glucose uptake and lactate release by astroglia. As astroglial uptake of glucose and subsequent lactate production contribute to neuronal metabolic activity and energy production, such findings suggest that, beside an effect on ceramide content, saturated fatty acids may result in detrimental changes in glucose metabolism further contributing to AD pathogenesis [180].

In subsequent studies it was demonstrated that soluble products released from PA-activated human astroglia activate neuronal N-SMase, increase ceramide levels, and provoke cell death in primary neuronal cultures. Namely, PA-induced activation of SPT and* de novo* synthesis of ceramides in astrocytes leads to production of proinflammatory cytokines TNF-*α* and IL-1*β*. After release in surrounding media, these soluble mediators activate SMases in neurons and increase ceramide levels, BACE1, and ultimately A*β* level [182].

## 4. Ceramides and Inflammation

Immune activation within the brain is classical feature of AD. Chronic neuroinflammation includes continuous activation of microglia and sustained release of inflammatory mediators, together with resulting oxidative and nitrosative stress [183, 184]. Following microglial activation numerous neurotoxic molecules including cytokines, chemokines, excitotoxic molecules, and reactive oxygen and nitrous species are released with detrimental consequences on neuronal survival. For example, A*β* induced the expression of inducible NO synthase (iNOS) and proinflammatory cytokines TNF-*α*, IL-1*β*, and IL-6 in mouse primary microglia [185]. As mentioned previously, some of these inflammatory mediators likely elicit production of ceramide via hydrolysis of sphingomyelin after SMase activation, whereas ceramide in turn could stimulate generation of ROS and initiate apoptotic cascade [54, 162]. The proinflammatory cytokine TNF-*α* acts as strong activator of N-SMase coupled to TNF receptor Type 1 (TNFR1) expressed on the surface of neuronal cells through the adaptor protein FAN [186]. In response to TNF-*α*, signalling through TNFR1 receptor induces phospholipase A2 activity, generation of arachidonic acid, and subsequent activation of N-SMase as arachidonic acid cascade produces 4-HNE and diverse ROS [162, 187]. Furthermore, activity of N-SMase preceded an accumulation of ROS by NADPH oxidase. It is known that NADPH oxidase-dependent irreversible damage to sphingosine kinase-1 inhibits neurite outgrowth of dorsal root ganglion neurons. Thus, TNF-*α*-induced N-SMase activation not only produces neurotoxic mediators such as ceramide and ROS, but also directly antagonizes neuronal survival. These findings suggest that a shift in ceramide metabolism in combination with oxidative stress likely exacerbates neuronal degeneration in AD and other CNS pathologies exhibiting inflammation [66]. Similarly, experiments performed on purified myelin from mouse brain showed that TNF-*α* upon release activates myelin-associated SMase resulting in ceramide production that might have deleterious effects on myelin integrity [188]. TNF-*α* also affects growth and viability of GT1-7 hypothalamic neurons through the ceramide-generating pathway [189]. The relationship between A*β* and oxidative stress in white matter involving cytokine and ceramide is also established. A*β* enhances TNF-*α*-induced expression of iNOS and nitrite generation in oligodendrocytes. A*β* and TNF-*α*, alone or in combination, increase activity of N-SMase leading to ceramide accumulation [190].

## 5. Sphingomyelin/Ceramide Pathway as a Therapeutic Approach in AD

Due to plethora of intrinsic factors affecting AD, it is generally assumed that novel pharmacological approaches in AD should rely on the simultaneous targeting of several steps in the early stages of the disease. Considering reports that intracellular accumulation of ceramides and neurotoxicity of A*β* can be prevented with inhibitors of ceramide production, it was suggested that sphingolipid metabolism might be a promising target for AD [27]. Pharmacological inhibition of sphingolipid biosynthesis may reduce A*β* production. Thus, neurons treated with conditioned media obtained from astroglia exposed to fatty acids and L-cycloserine, the SPT inhibitor, displayed attenuated increase in A*β* levels [180]. Furthermore, subcutaneous administration of L-cycloserine to the aforementioned early-onset mouse model of AD effectively reduced cortical levels of SPT, together with reduced ceramide levels, and downregulation of cortical A*β* production [181].

Minocycline, a semisynthetic second-generation tetracycline derivative that effectively crosses the blood-brain barrier, also exerts beneficial effects against sphingomyelinase/ceramide-induced neurotoxicity via antioxidative and antiapoptotic mechanisms [191]. In particular, it showed neuroprotective effects in rat cortical cultures against toxicity induced by bacterial SMase and C2-ceramide, by enhancing Bcl-2 expression via activation of the NO/cGMP/PKG pathway and direct induction of PKG-1, in association with increased expression of thioredoxin-1 [65]. Minocycline was also effective in attenuating A*β*-induced neuronal cell death and deficits in learning and memory in A*β*-infused rats [192].

Besides acting on A*β* pathology, ceramides might affect hyperphosphorylation of tau by modulating PP2A activity, the principal phospho-tau phosphatase. Ceramides activate PP2A* in vitro* and in rat brain, and inhibitor 2 of PP2A (I2PP2A) was identified as a ceramide-binding protein that through its specific interactions with ceramide prevents inhibition of PP2A activity* in vitro* [193–195]. The enhancement of the PP2A activity is considered promising as a next generation of the AD therapeutic target [196]. Lithium was found effective against ceramide-induced apoptosis via inhibition of PP2A activity. Ceramide-induced activation of PP2A involved methylation of PP2A C subunit, which was suppressed by lithium. Lithium also exhibited antiapoptotic effect by inhibiting Bcl-2 dephosphorylation and caspase-2 activation [197]. It is also suggested that PARP-1 inhibitors and modulators of sphingosine-1-phosphate receptors may represent a promising avenue in future therapeutic approaches [70].

## 6. Conclusions

Ceramides affect various aspects of neuronal physiology determining cell differentiation, growth, survival, senescence, and apoptosis. Postmortem analyses of AD brains reveal alterations of brain lipid profile and disturbed ceramide metabolism indicating that preservation of ceramide homeostasis is a prerequisite for normal brain functioning. Moreover, increased ceramide levels are found in patients with mild and moderate symptoms, suggesting that alterations in ceramide metabolism occur in the early stages of the disease. These findings pointed out that ceramides exert important roles in the AD onset and progression. Numerous evidences demonstrated that ceramides are detrimentally associated with several pathological aspects of AD, including A*β* accumulation, tau hyperphosphorylation, ROS generation, mitochondrial dysfunction, deregulation of intracellular signalling pathways, initiation of apoptosis, and neurodegeneration.

Of particular interest is the critical role of ceramides in mediating the production of A*β* and their link to oxidative stress. Ceramides increase A*β* levels by reinforcing APP processing via BACE1 stabilization. As soluble and fibrillar forms of A*β* can activate both neutral and acidic SMases through increased ROS accumulation via NADPH oxidase activation and GSH depletion, initial ceramide formation in a vicious cycle leads to more ceramide accumulation. In addition to ceramide increase, oxidative stress is also one of the earliest events in AD. Enhanced generation of free radicals strongly contributes to toxic effects of both ceramides and A*β*, ultimately affecting neuronal functioning and survival. Membrane oxidative stress seems to be particularly relevant for AD, as accumulation of HNE, the toxic end-product of lipid peroxidation, is closely related to the severity of neurodegenerative changes.

In spite of major research efforts, there is still no effective AD treatment. A comprehensive understanding and identification of molecular and biochemical alterations in ceramide metabolic pathways and insight into their influence on the pathophysiological processes during progression of AD are indispensable for successful development of novel therapeutic interventions. Strategies aimed at reducing ceramide levels have the potential to prevent A*β* burden and slow down progression of the disease. Further studies* in vivo* are needed to establish proof of concept, particularly in combination with other targets, such as those directed at detoxification of ROS and preservation of redox homeostasis.
